# Primary extramammary invasive Paget’s vulvar disease: what is the standard, what are the challenges and what is the future for radiotherapy?

**DOI:** 10.1186/s12885-016-2622-5

**Published:** 2016-07-29

**Authors:** Maria Tolia, Nikolaos Tsoukalas, Chrisostomos Sofoudis, Constantinos Giaginis, Despoina Spyropoulou, Dimitrios Kardamakis, Vasileios Kouloulias, George Kyrgias

**Affiliations:** 1 Department of Radiotherapy, University of Thessaly, School of Health Sciences, Faculty of Medicine, Biopolis, Larissa, 411 10 Greece; 2 Oncology Clinic, 401 General Military Hospital, Mesogeion 138 & Katehaki, 115 25 Athens, Greece; 3 LITO Obstetrics, Gynaecology & Surgery Centre, 7-13 Mouson Str, Psychiko-Athens, 154 52 Greece; 4 Department of Food Science and Nutrition, University of the Aegean, Myrina, Lemnos 814 40 Greece; 5 Department of Radiation Oncology, University of Patra Medical School, Patra, 265 04 Greece; 6 Second Department of Radiology, Radiation Therapy Oncology Unit, University Hospital of Athens “ATTIKON”, Rimini 1, Haidari, 124 64 Athens, Greece

**Keywords:** Radiotherapy, Extramammary invasive Paget’s disease, Vulva

## Abstract

**Background:**

Primary invasive Extramammary Paget’s vulvar disease is a rare tumor that is challenging to control. Wide surgical excision represents the standard treatment approach for Primary invasive Extramammary Paget’s vulvar disease. The goal of the current study was to analyze the appropriate indications of radiotherapy in Primary invasive Extramammary Paget's vulvar disease because they are still controversial.

**Discussion:**

We searched the Cochrane Gynecological Cancer Group Trials Register, Cochrane Register of Controlled Trials (CENTRAL), MEDLINE and EMBASE database up to September 2015. Radiotherapy was delivered as a treatment in various settings: i) Radical in 28 cases (range: 60–63 Gy), ii) Adjuvant in 25 cases (range: 39–60 Gy), iii) Salvage in recurrence of 3 patients (63 Gy) and iv) Neoadjuvant in one patient (43.3 Gy). A radiotherapy field that covered the gross tumor site with a 2–5 cm margin for the microscopic disease has been used. Radiotherapy of the inguinal, pelvic or para-aortic lymph node should be considered only for the cases with lymph node metastases within these areas.

**Summary:**

Radiotherapy alone is an alternative therapeutic approach for patients with extensive inoperable disease or medical contraindications. Definitive radiotherapy can be used in elderly patients and/or with medical contraindications. Adjuvant radiotherapy may be considered in presence of risk factors associated with local recurrence as dermal invasion, lymph node metastasis, close or positive surgical margins, perineal, large tumor diameter, multifocal lesions, extensive disease, coexisting histology of adenocarcinoma or vulvar carcinoma, high Ki-67 expression, adnexal involvement and probably in overexpression of HER-2/neu. Salvage radiotherapy can be given in inoperable loco-regional recurrence and to those who refused additional surgery.

## Background

Primary Extra-mammary Paget’s disease (EMPVD) is a rare form of skin cancer. Vulva is one of the most common sites of involvement accounting for approximately 1 % of all cases [1, 2]. It usually occurs in postmenopausal women between 50 and 80 years of age [3]. EMPVD originates from basal epidermal cells and extends beyond the apparent edges of the lesion. Surgery is the standard treatment [4], but even with the use of more radical procedures, local recurrence remains common (15–61 % of cases) due to microscopic extension, positive margins, and multicentric disease [3, 5].

Despite the high recurrence rate, there is little consensus regarding the value of radiotherapy (RT) in EMPVD. Most prior studies have been derived from limited cases of single institution experience. The use of RT, alone or in combination with surgery in the management of EMPVD, warrants further investigation. The aim of this review was to evaluate the potential benefit of RT for EMPVD.

## Materials and methods

The key words used for the search were: «Extramammary Invasive Paget’s disease», «Paget’s vulvar disease», «Radiotherapy» and synonyms). A literature review was performed based on database search in: Cochrane Gynecological Cancer Group Trials Register, Cochrane Register of Controlled Trials (CENTRAL), MEDLINE and EMBASE up to September 2015.

**Fig. 1:**
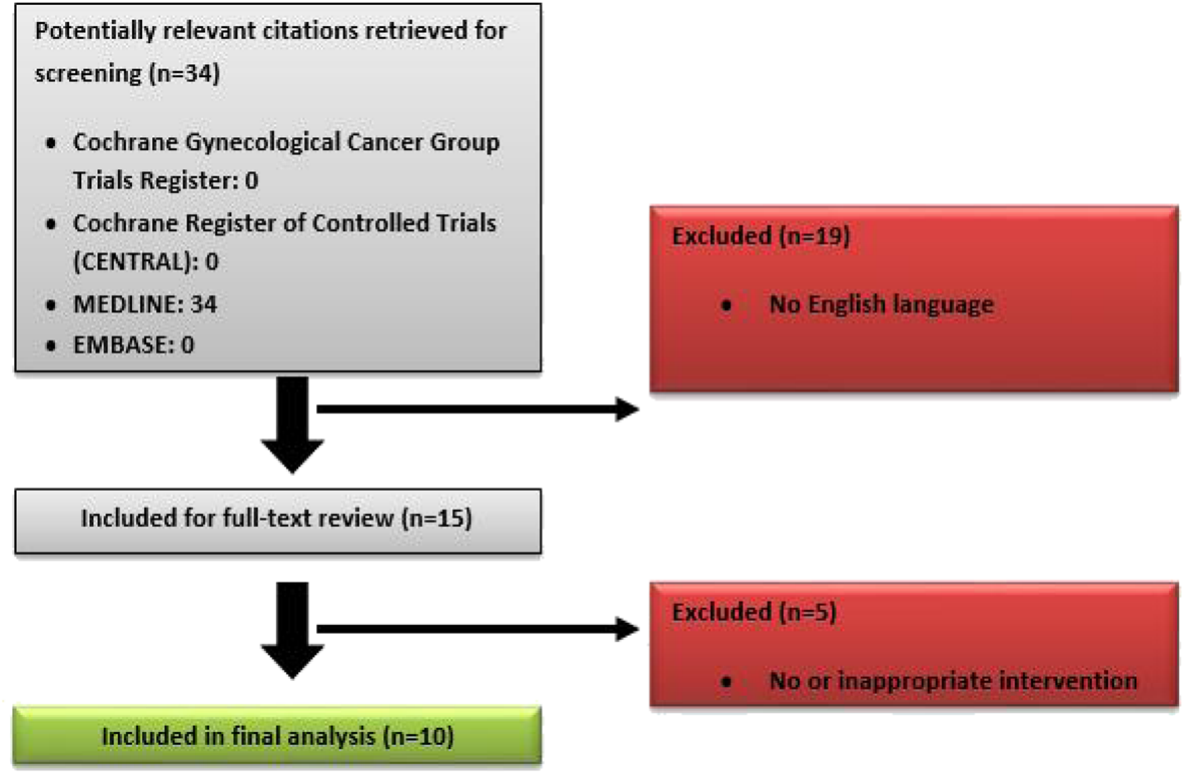
Literature search results and study selection

The exclusion criteria were: 1) pre-clinical studies, 2) not English language and 3) studies with no or inappropriate intervention (Fig. 1)

.

The search of the literature identified 34 papers. Twenty seven publications were excluded after the study of their summaries, as they are not related to «Primary Extramammary Invasive Paget’s Vulvar Disease: what is the standard, what are the challenges and what is the future for radiotherapy?».

**Fig. 2:**
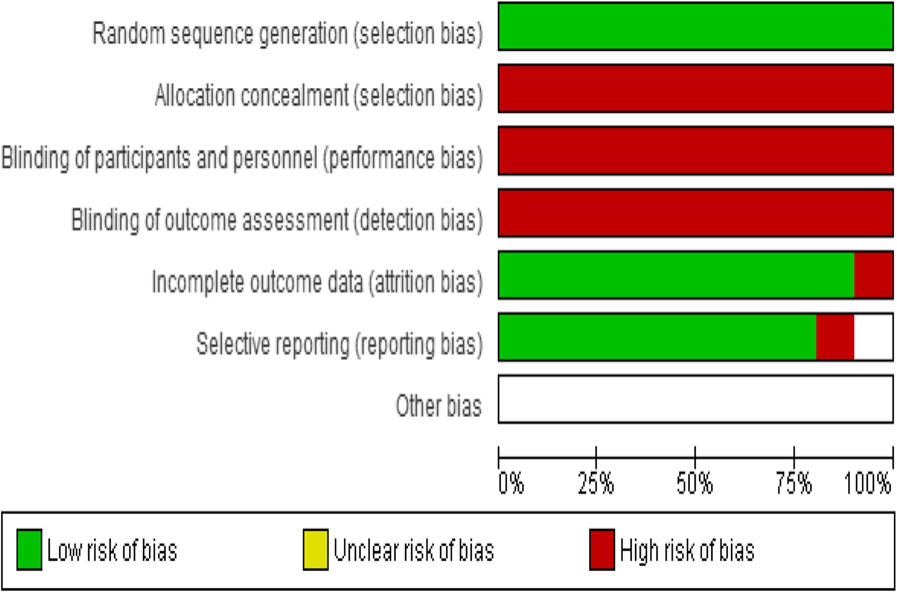
Risk of Bias graph (Cochrane Collaboration)

**Fig. 3:**
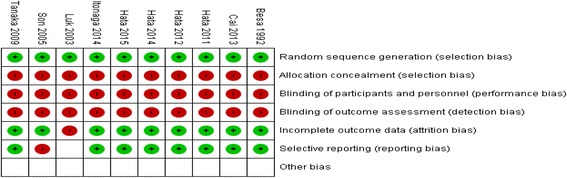
Risk of Bias summary (Cochrane Collaboration)

The Quality rating of included studies was based on the COCHRANE RISK OF BIAS TOOL. The quality of the studies was medium (Figs. 2 and 3). All of the studies were small in sample size. In general, study design was attributed to the rarity of the condition.

## Results

**Fig. 4:**
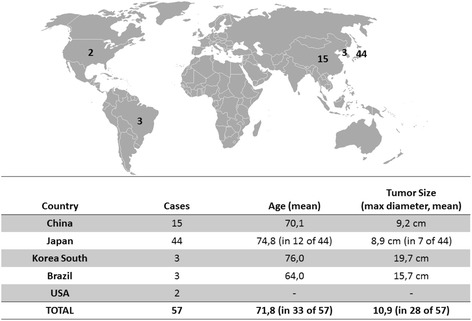
Geographical distribution and characteristics of cases

From a review of all published studies up to September 2015, a total of 57 invasive EMPVD patients underwent RT (Tables 1 and 2). From the total of the read papers, we created an infographic that contains: 1) Geographical distribution of cases 2) The patient’s characteristics related to the age at the time of diagnosis. 3) The characteristics related to the diameter of the tumor before radiotherapy (Fig. 4).

**Table 1:** Clinical outcome of Radiotherapy in selected studies

Author and year publication	*N*	Median follow up (mo)	RT intent	RT total dose (Gy)	Toxicity	DFS (mo)	OS (mo)	LC % (mo)	Conclusions
Besa et al. 1992 [6]	2	12–66	1: Definitive	44–64	-	-	-	-	Dose greater than 50 Gy in who is medically unfit for surgery and for organ preservation could be indicated.
			1: Post-operative						
Luk et al. 2003 [7]	1	14–174	1: Post-operative	60 TB + 32 IN	Acute: confluent wet desquamation, enteritis grade 2	10	15	100 (24)	The results confirmed the useful role of radiotherapy in the management of extramammary Paget’s disease.
					Late: ≤ grade 2 skin atrophy				
Son et al. 2005 [8]	3	6-96	2: Definitive	A) 55.8 1ary	Acute: Dermatitis grade 2–3	A)12	A) -	100 A)(24)	RT is of benefit in some selected cases of EMPD.
						B)-	B)-	B)(6)	
						C)96	C)-	C)(96)	
					Late: ≤ grade 2 skin atrophy				
				B) 81.6 1ary + 45.6 IN					
			1: Post-operative						
				C) 55.8 TB					
Tanaka et al. 2009 [9]	2	18-84	2: Definitive	60	-	A) 18	A)-	100 A)(18)	EMPD is an uncommon neoplasm without any effective treatment.
						B) 84	B)-	B)(84)	
Hata et al. 2011 [10]	12	8–133	4: Definitive	45–70.2 Gy (60)	Acute: ≤ Grade 3 hematologic toxicity, dermatitis, cystitis, enteritis, urethritis	24	(100 %) 24 mo	100 % (2–9)	RT is safe and effective for patients with EMPD. It appears to contribute to prolonged survival as a result of good tumor control.
			8: Post-operative						
					Late: telangiectasia				
Hata et al. 2012 [11]	7	18–150	7: Definitive	59.4–70.2	Acute: ≤ Grade 3 hematologic toxicity, dermatitis, cystitis, enteritis, urethritis	58 % (36)	92 % (36)	71 % (36)	Radiation therapy is effective and safe, and appears to offer a curative treatment option for patients with EMPD.
						46 % (60)	79 % (60)	(60)	
					Late: ≤ Grade 3 telangiectasia				
Cai et al. 2013 [2]	5	7–169	1: Pre-operative	57–60	Acute, Late: Acceptable ≤ Grade 3	-	70.8 mo (Invasive) 21.3 mo (associated with adnexal adenocarcinoma)	-	Intraepithelial EMPDV accounted for the majority of primary cases and had a better prognosis.
			4: Post-operative						
									Surgical excision was the standard curative treatment for EMPDV. Radiotherapy was an alternative choice
									for patients with medical contradiction or surgical difficulties. Postoperative radiotherapy could be considered
									in cases with positive surgical margin or lymph node metastasis. Recurrence was common and repeated excision was often necessary.
Hata et al. 2014 [12]	14	2–174	10: Definitive	45–80.2 (60)	Acute: ≤grade 2 hematologic toxicities, dermatitis, colitis, cystitis	54 % (36)	62 % (60)	88 % (36)	Radiation therapy is safe and effective for patients with EMPD. It appeared to contribute to prolonged survival owing to good tumor control, and to be a promising curative treatment option.
								46 % (60)	
			4: Post-operative						
					Late: ≤ Grade 3 telangiectasia				
Itonaga et al. 2014 [15]	7	Median 71.4	2: Definitive	50	Acute, Late: Acceptable ≤ Grade 3	91.7 % (60)	84.3 % (60)	91.7 % (60)	Radiotherapy yielded good local control and survival, which suggests that it was effective for patients with EMPD and in particular medically inoperable EMPD.
			2: Post-operative						
			3: after surgical relapse						
Hata et al. 2015 [16]	4	2–109	4: Post-operative	45–64.8	Acute: ≤ grade 2 dermatitis, grade 1 colitis and cystitis	92 % (36)	92 % (36)	100 % (38)	Postoperative radiation therapy is safe and effective in maintaining local control in patients with EMPD.
						71 % (60)	62 % (60)		
					Late: grade 1 telangiectasia				

*Abbreviations*: *N* number of patients, *DFS* Disease free survival, *OS* Overall survival, *LC* local control, *1ary* Primary Disease, *TB* tumor bed, *IN* Inguinal Nodal Areas

**Table 2:** Negative prognostic factors as they were evaluated by each study

Authors/Prognostic factors	Close or positive surgical margins	Dermal invasion	Lymph node metastasis	Adnexal involvement	Tumor size	Coexisting histology of adenocarcinoma or vulvar carcinoma	Perineal involvement	Stage	Multifocality
Besa et al. 1992 [6]	+			+	+	+	+	+	
Luk et al. 2003 [7]					+	+	+	+	
Son et al. 2005 [8]					+	+			
Tanaka et al. 2009 [9]	+	+	+						
Hata et al. 2011 [10]	+	+	+						
Hata et al. 2012 [11]		+	+		+				
Cai et al. 2013 [2]	+	+	+	+		+			+
Hata et al. 2014 [12]		+	+		+				
Hata et al. 2015 [16]		+	+		+

Besa et al. [6] reported the clinical course of 2 EMPVD patients that underwent definitive RT. They were treated to a total dose of 44–64 Gy in 23–28 fractions. The investigators concluded that dose greater than 50 Gy to those who are medically unfit for surgery and for organ preservation could be indicated. For the cases of EMPVD mixed with adenocarcinoma, the use of adjuvant postoperative RT in doses greater than 55 Gy should be considered because of the high risk of local recurrence after surgery alone.

Luk et al. [7] delivered adjuvant RT (60 Gy) in one EMPVD patient with stage III vulvar carcinoma. RT techniques included anteroposterior opposed photon fields to pelvis and vulva, then electron boost to right vulva with 10 MeV electron field. The patient had a very good local control but she died at 15 months after RT of distant metastasis.

Son et al. [8] treated 3 patients with EMPVD. Two of them received definitive RT. The first case received 55.8 Gy to the primary and the second received 81.6 Gy to the primary and 45.6 Gy to the inguinal nodal areas. The last case received adjuvant RT to a total dose of 55.8 Gy. The RT fields encompassed 2–3 cm radially the clinically visible disease. In all cases 6 MV photons and 9 MeV electrons were used.

Tanaka et al. [9] treated 2 EMPVD patients with definitive RT. Both received 60 Gy with electrons. The patients were also treated with CO2 laser therapy and Mohs micrographic surgery respectively because of local recurrence. They had a complete response and a disease free survival of 3 and 7 years respectively.

Hata et al. [10] underwent RT to 12 EMPVD patients to the primary, pelvic and inguinal lymph nodes. The authors used anteroposterior opposed ports with 4–6-MV X-rays, followed by a local RT boost to the gross tumor site using 7–13 or 6–15 MeV electrons. The RT fields included the gross tumor or the tumor bed (including the positive surgical margin), with a 2–5-cm margin. A bolus with a 5–10-mm water-equivalent thickness was used to compensate for the skin surface dose. Total doses of 45–70.2 Gy (median 60) were delivered with a fraction size of 1.8–2.2 Gy. A total dose of 59.4–70.2 Gy (median 60.6) was given to the gross primary and metastatic inguinal lymph nodes. In case of positive surgical margin the patients received a dose of 45–64.8 Gy (median 50) to the tumor bed. The 2- and 5-year local progression-free rates were 91 and 84 %, respectively. The overall and cause-specific survival rates were 100 % for both at 2 years and 53 % and 73 % at 5 years, respectively. None of the patients who received RT to the local lymph node regions developed a recurrence and this suggests that inclusion of the draining groin lymph nodes into the RT field might be important in optimizing treatment outcome. No therapy-related Grade 3 or greater toxicity was observed.

Hata et al. [11] treated seven EMPVD patients with definitive RT therapy. Two cases were irradiated to the local tumor site and to the regional (pelvic and inguinal) lymph node area through antero-posterior opposed ports with 4–6 MV X-rays. This was followed by a local RT boost to the gross tumor site using 6–13 MeV electrons. The remaining five patients underwent local RT only to the tumor site, using 4–14 MV X-rays or 6–15 MeV electrons. RT fields were set up to include the primary lesion with a 2–5 cm margin. The RT field was reduced after doses of 44–45 Gy, and total doses of 59.4–70.2 Gy were subsequently delivered to the gross tumors, including enlarged metastatic inguinal lymph nodes. The overall local control rate was 71 % at both 3 and 5 years. The disease-free, and overall survival rates in all patients were 58, and 92 % at 3 years, and 46, and 79 % at 5 years, respectively. No therapy-related toxicities of grade 3 or greater were observed.

Cai et al. [2] delivered RT in five patients (1: preoperative, 4: postoperative). The radiation fields were set up according to the tumor size with a 2–3 cm margin. The total dose ranged between 57–60 Gy. During a follow-up of 7–169 months, the median overall survival was 70.8 months in invasive cases and 21.3 months in cases with adnexal adenocarcinomas. A higher local recurrence was associated with the presence of a positive margin, with associated adnexal adenocarcinomas histology, and presence of dermal invasion.

Hata et al. [12] delivered RT in a total of 14 EMPVD patients. The patients with regional lymph node metastases underwent RT with 4–15 MV X-rays to the local tumor site and the lymph nodal areas. A local RT boost to the gross disease or involved single node (45–80.2 Gy, median 60 Gy) was delivered using 6–13 MeV electrons. The patients without nodal involvement received RT to the tumor, using 4–15 MV X-rays or 6–15 MeV electrons. The prophylactic dose delivered to the regional lymph node area was 41.4–50.4 Gy. RT fields included the tumor, or the tumor bed with a 2–5 cm margin. A bolus with a 5–10 mm water equivalent thickness was used to compensate for the surface dose in the area of tumor. At a median follow-up period of 41 months, the local control was 88 and 46 % in 3 and 5 years respectively. The DFS rate was 54 % in 3 years and the overall survival was 62 % in 5 years. The authors [13, 14] found that tumor dermal invasion and presence of regional lymph node metastasis were the most significant prognostic factors for both distant metastasis and survival. Due to the fact that it is usually difficult to keep the bolus into close contact with the vulva, the tumor surface may have possibly been covered with lower RT doses than those prescribed, due to this technical difficulty. The authors [12–14] also concluded that there was no significant difference in the local recurrence rate between EMPVD patients treated with margins of >2 and ≤2 cm.

Itonaga et al. [15] studied the outcome of 7 EMPVD patients. All patients were treated with curative intent and they received a median dose of 50 Gy. The patients achieved complete response within the irradiated volume during a median follow-up period of 71.4 months. The 5-year locoregional progression-free survival and overall survival were 91.7 and 84.3 %, respectively.

Hata et al. [16] delivered adjuvant post-operative RT in 4 EMPVD patients. A median total dose of 59.4 Gy (range, 45–64.8 Gy) was delivered to the tumour bed with a margin of at least 2 cm. A bolus with a 5–10-mm water equivalent thickness was used to compensate for the surface dose. At a median follow-up period of 38 months all patients had local control. The OS and CSS rates were both 92 % in 3 years, and 62 and 71 % in 5 years, respectively. No therapy-related acute or late toxicities of grade ≥ 3 were observed.

Karam et al. [17] used the data derived from the Surveillance, Epidemiology and End Results (SEER) program, and undertook a retrospective analysis of the treatments approaches and outcome of 92 EMPVD patients. The authors found a significantly worse disease specific survival (DSS) associated with the use of radiation therapy even when combined with site directed surgery. The authors found that patients who underwent surgery alone had a most favorable prognosis with a mean DSS compared to patients who did not undergo surgery or RT (346.8 vs. 255.1 months, 95 % CI 221.1–289.2, *p* = 0.002), to patients who received RT alone (mean DSS 143.4 months, 95 % CI 119.2–167.5, *p* = 0.004) and with patients who underwent surgery and RT (mean DSS 120.6 months, 95 % CI 93.6–147.6, *p* < 0.001). These outcomes may be explained by the fact that the use of RT was associated with locally advanced stage or recurrent disease. Interpretation of this analysis is difficult because radiation fields, doses and schedules were not standardized between patients.

Several studies [13, 14, 18–20] have reported various prognostic factors that predict the poorer outcome of EMPVD, including dermal invasion, lymph node metastasis, stage [13, 14], tumor coexisting histology of adenocarcinoma [2] or vulvar carcinoma, positive surgical margins [2], HER-2/neu overexpression [19] and high expression of Ki-67 [20].

## Conclusions

Wide surgical excision remains the standard therapeutic approach of EMPVD although this may not always demonstrate acceptable rates of local control [2]. Since the lesions are often multifocal and the margins are irregular, involvement of microscopic margins occurs in approximately 40 –75 % of patients following surgical excision [2]. Postoperative RT could be considered in presence of cases with positive surgical margin, lymph node metastasis, multifocal disease, associated adnexal adenocarcinomas.

However, surgery is sometimes not possible, because many patients are elderly, and complete excision can be difficult owing to the tumor location. Definitive RT as a first-line treatment used only in a small number of patients could be an alternative choice in case of medical contraindication or surgical difficulties because of extensive disease, tumor location and perineal involvement [5].

The optimal radiation dose for EMPD has not been established [21]. With the limited data available in the studies, it is not possible to form any conclusions with regards to local control as a treatment and disease-free survival. Moreover, in practice RT doses, fields, techniques and fractionation may vary widely [22].

A radiation field that encompasses the gross tumor volume with a 2–5 cm margin radially has been used in most patients [2]. RT of the inguinal, pelvic or para-aortic lymph nodal chains can be considered only for the cases with lymph node metastases within these areas [2, 10].

## References

[1] Lam C, Funaro D (2010). Extramammary Paget’s disease: summary of current knowledge. Dermatol Clin.

[2] Kanitakis J (2007). Mammary and extramammary Paget’s disease. J Eur Acad Dermatol Venereol.

[3] Cai Y, Sheng W, Xiang L, Wu X, Yang H (2013). Primary extramammary Paget’s disease of the vulva: the clinicopathological features andtreatment outcomes in a series of 43 patients. Gynecol Oncol.

[4] Bakalianou K, Salakos N, Iavazzo C (2008). Paget’s disease of the vulva. A ten-year experience. Eur J Gynaecol Oncol.

[5] Shaco-Levy R, Bean SM, Vollmer RT (2010). Paget disease of the vulva: a study of 56 patients. Eur J Obstet Gynecol Reprod Biol.

[6] Besa P, Rich TA, Delclos L, Edwards CL, Ota DM, Wharton JT (1992). Extramammary Paget’s disease of the perineal skin: role of radiotherapy. Int J Radiat Oncol Biol Phys.

[7] Luk NM, Yu KH, Yeung WK, Choi CL, Teo ML (2003). Extramammary Paget’s disease: outcome of radiotherapy with curative intent. Clin Exp Dermatol.

[8] Son SH, Lee JS, Kim YS (2005). The role of radiation therapy for the extramammary paget’s disease of the vulva; experience of 3 cases. Cancer Res Treat.

[9] Tanaka VD, Sanches JA, Torezan L, Niwa AB, Festa NC (2009). Mammary and extramammary Paget’s disease: a study of 14 cases and the associated therapeutic difficulties. Clinics (Sao Paulo).

[10] Hata M, Omura M, Koike I (2011). Role of radiotherapy as curative treatment of extramammary Paget’s disease. Int J Radiat Oncol Biol Phys.

[11] Hata M, Koike I, Wada H (2012). Definitive radiation therapy for extramammary Paget’s disease. Anticancer Res.

[12] Hata M, Koike I, Wada H (2014). Radiation therapy for lymph node metastasis from extramammary Paget’s disease. J Eur Acad Dermatol Venereol.

[13] Hata N, Yamada M, Hirano T (2008). Extramammary Paget’s disease: treatment, prognostic factors and outcome in 76 patients. Br J Dermatol.

[14] Hata M, Koike I, Wada H (2014). Radiation therapy for extramammary Paget’s disease: treatment outcomes and prognostic factors. Ann Oncol.

[15] Itonaga T, Nakayama H, Okubo M (2014). Radiotherapy in patients with extramammary Paget’s disease: our own experience and review of the literature. Oncol Res Treat.

[16] Hata M, Koike I, Wada H (2015). Postoperative radiation therapy for extramammary Paget’s disease. Br J Dermatol.

[17] Karam A, Dorigo O (2012). Treatment outcomes in a large cohort of patients with invasive Extramammary Paget’s disease. Gynecol Oncol.

[18] Parker LP, Parker JR, Bodurka-Bevers D (2000). Paget’s disease of the vulva: pathology, pattern of involvement, and prognosis. Gynaecologic Oncology.

[19] Richter CE, Hui P, Buza N (2010). HER-2/NEU overexpression in vulvar Paget disease: the Yale experience. J Clin Pathol.

[20] Aoyagi S, Akiyama M, Shimizu H (2008). High expression of Ki-67 and cyclin D1 in invasive extramammary Paget’s disease. J Dermatol Sci.

[21] Choi Y, Park W, Lee J, Cho EY, Moon GH (2014). Aggressive clinical course of extramammary Paget disease after radiotherapy. Radiat Oncol J.

[22] Edey KA, Allan E, Murdoch JB, Cooper S, Bryant A (2013). Interventions for the treatment of Paget’s disease of the vulva. Cochrane Database Syst Rev.

